# Acute blood pressure responses after different isometric handgrip protocols in hypertensive patients

**DOI:** 10.6061/clinics/2018/e373

**Published:** 2018-10-05

**Authors:** Gustavo O Silva, Breno Q Farah, Antonio H Germano-Soares, Aluísio Andrade-Lima, Fabio S Santana, Sérgio LC Rodrigues, Raphael M Ritti-Dias

**Affiliations:** I Universidade de Pernambuco, Recife, PE, BR; II Departamento de Educacao Fisica, Universidade Federal Rural de Pernambuco, Recife, PE, BR; III Departamento de Educação Física, Universidade Federal de Sergipe, SE, BR; IV Programa de Pos-graduacao em Ciencias da Reabilitacao, Universidade Nove de Julho, Sao Paulo, SP, BR

**Keywords:** Exercise, Blood Pressure, Hypertension

## Abstract

**OBJECTIVE:**

The present study analyzed blood pressure responses after a single session of isometric handgrip exercise performed with different volumes and intensities by patients with hypertension.

**METHODS:**

This randomized crossover trial submitted 12 hypertensive patients (58±5 years old) to four isometric handgrip exercise sessions in a random order: 4 x 2 min at 30% of the maximal voluntary contraction (S30%); 4 x 2 min at 50% of the maximal voluntary contraction (S50%2min); 4 x 3 min at 30% of the maximal voluntary contraction (S30%3min); and a control session. The systolic and diastolic blood pressure, heart rate, and rate-pressure product were measured pre- and post-exercise (30^th^ min).

**RESULTS:**

No significant changes were observed in cardiovascular variables after any session (*p*>0.05 for all comparisons). Similarly, individual analyses revealed heterogeneity in the responses, including increases in blood pressure observed in some sessions. Patients with reduced blood pressure after an isometric handgrip exercise session exhibited a higher body mass index, diastolic blood pressure and heart rate (*p*<0.05). They also tended to be younger (*p*=0.07).

**CONCLUSION:**

Isometric handgrip exercise performed with different intensities and volumes did not reduce the blood pressure of hypertensive patients.

## INTRODUCTION

Hypertension affects more than 1 billion adults worldwide 1, and it remains one of the most significant risk factors for cardiovascular diseases 2, accounting for 13% of total deaths worldwide 3. Isometric handgrip (IHG) training can reduce blood pressure in hypertensive patients 4-9. Therefore, the American Heart Association has classified IHG training as a potential therapy for these patients. However, further studies are needed (Level of Evidence C) 10.

Studies that analyzed the acute effects of isometric exercise observed increased 11,12, maintenance 13 or decreased 14 blood pressure after exercise. These discrepancies may be attributed to the exercise protocols, which used different volumes and intensities [3min at 30% of the maximal voluntary contraction – MVC 11; 30 min at 5%, 10% or 15% of the MVC 12; 4 x 5 contractions of a 10-second duration at 50% of the MVC 13; and 4 x 2 min at 30% of the MVC 14]. However, whether the modulation of volume and intensity affects cardiovascular responses after IHG training, especially in hypertensive patients, remains unknown 9. This information is important because acute cardiovascular responses to exercise predict chronic adaptations to exercise training 15. Therefore, a better understanding of cardiovascular responses to a single IHG session is clinically relevant.

Cardiovascular responses to exercise also exhibit intra- and inter-subject heterogeneity 16. Costa et al. 17 used an aerobic exercise model and observed that some of the patients exhibited greater blood pressure reductions after high intensity aerobic exercise, and the other patients exhibited greater responses after moderate intensity exercise. This variability may also exist after different IHG protocols and should be analyzed.

Therefore, the present study examined blood pressure changes following IHG protocols with different volumes and intensities in hypertensive patients.

## METHODS

### Study design and sample

This randomized crossover trial included hypertensive patients of both sexes who were participating in an IHG program for six weeks. The following inclusion criteria were used: a) use of anti-hypertensive medications b) >18 years old, c) absence of diabetes or cardiovascular disease, d) no limitations for performing IHG, and e) no other regular physical activity programs. The ethics committee of the University of Pernambuco approved the study protocol, which was performed in compliance with the Declaration of Helsinki and Brazilian National Research Ethics System Guidelines.

### Pre-experimental procedures

#### Clinical data

Demographic data, medical history and medication use were self-reported. Height and weight were measured using standardized protocols, and the body mass index was calculated.

#### Maximal voluntary contraction

Participants were familiarized with testing procedures prior to data collection, and 1-2 additional familiarization sessions were performed to ensure satisfactory performance. Participants were seated with their elbow flexed at 90°, and a MVC was performed 3 times using their dominant hand and a handgrip dynamometer (Zhongshan Camry Electronic Co. Ltd., Zhongshan Guangdong, China). The highest recorded trial was recorded as the MVC 18. Participants were encouraged to breathe spontaneously without performing the Valsalva maneuver during contraction. All procedures were performed according to the recommendations of the American Society of Hand Therapists 19, and the intraclass correlation coefficient of the MVC ranged from 0.986 (non-dominant hand) to 0.989 (dominant hand) 18.

### Experimental procedures

Each experimental session was performed at the same time of day (10:00 a.m. to 12 p.m.) and with a 48-hour interval between sessions to avoid circadian influence and residual effects of exercise, respectively. The order in which the patients performed each experimental session was determined using simple randomization (www.randomizer.org). The experimental session was performed as follows: a) four sets of 2-min isometric contractions at 30% MVC (S30%2min); b) four sets of 2-min isometric contractions at 50% MVC (S50%2min); c) four sets of 3-min isometric contractions at 30% MVC (S30%3min); and, d) remaining seated without performing the exercise (control session - CS). An interval of 1 min between sets was used in each session, except for the S30%3min session, which did not include a rest interval between sets to standardize the duration of the experimental sessions. Patients were instructed to eat a light meal and avoid exercise, alcohol and caffeine ingestion for 24 hours prior to the experiment. All sessions were performed in a temperature-controlled room, free from noises and external distractions.

**Figure 1:**
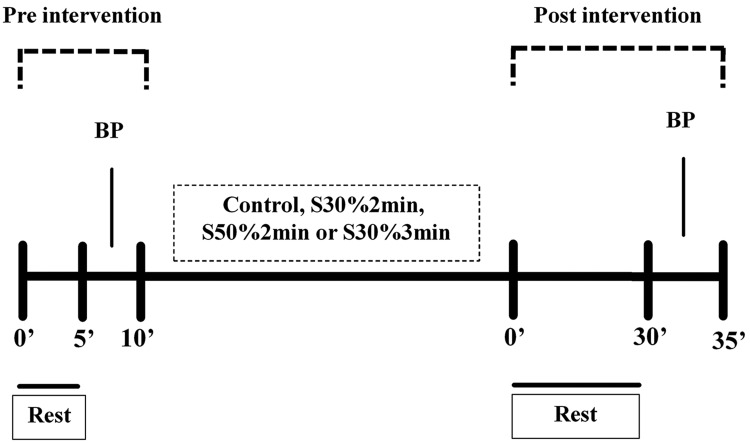
Design of the experimental sessions. BP **-** blood pressure measurement.

Figure 1 shows the design of the experimental sessions. Patients remained seated for 10 min (pre-intervention period) prior to each experimental session. Blood pressure and heart rate values were measured after a 5-min rest. Participants performed the corresponding experimental session (∼12 min in duration) in a seated position with the elbow flexed at 90° and holding the handgrip. All patients were supervised during the protocol to avoid inappropriate execution of the exercise. Patients rested for 30 min after the intervention (post-intervention period), and blood pressure and heart rate values were measured.

### Cardiovascular measurement

The systolic blood pressure, diastolic blood pressure and heart rate were measured using the Omron HEM 742 (Sao Paulo, Sao Paulo – Brazil). Participants remained in a seated position for at least 5 min in a temperature-controlled room for these measurements. The blood pressure was measured at least three consecutive times in the right arm, until the values were within 4 mmHg of the previous value, with 1-min intervals between measurements. The average of the last two measurements was used, which followed the procedures of VII Brazilian Guidelines on Hypertension 20. The rate-pressure product was calculated as heart rate *systolic blood pressure. Reductions of 4 mmHg or greater in systolic or diastolic blood pressure were considered clinically relevant, as previously suggested 21.

### Statistical analysis

The sample size calculation was performed using G*Power (version 3.1.0). The minimum sample size required was 12 patients per session considering an effect size of 0.25, a power of 80% and an alpha error of 5%. The normality and homogeneity of variance of the data were determined using the Shapiro-Wilk and Levene's tests, respectively. Comparisons of pre-intervention blood pressure, heart rate, and rate-pressure product among protocols were performed using one-way ANOVA. Two-way ANOVA for repeated measures was used for pre- to post-blood pressure, heart rate, and rate-pressure product comparisons among protocols, using time and protocol as factors. We compared the CS, S30%2min, and S50%2min sessions to examine the influence of exercise intensity and exercise volume. We used Chi-square and paired t-tests to examine whether the clinical characteristics differed between patients who did or did not exhibit reductions ≥4 mmHg in systolic or diastolic blood pressures in at least one exercise session. Individual analyses were performed by calculating the net effect (changes after each exercise session minus the changes in the CS for each patient) of systolic and diastolic blood pressures. All analyses were performed using STATISTICA (version 7.0) and SPSS (version 21.0), and a value of *p*≤0.05 was considered statistically significant.

## RESULTS

Twelve hypertensive patients (16.6% men) fulfilled the study criteria and participated in the study. Table 1 shows their clinical characteristics.

Table 2 shows the comparisons of the blood pressure, heart rate, and rate-pressure product in the pre-intervention period among experimental sessions. No difference was found in any of the parameters (*p*>0.05).

**Figure 2:**
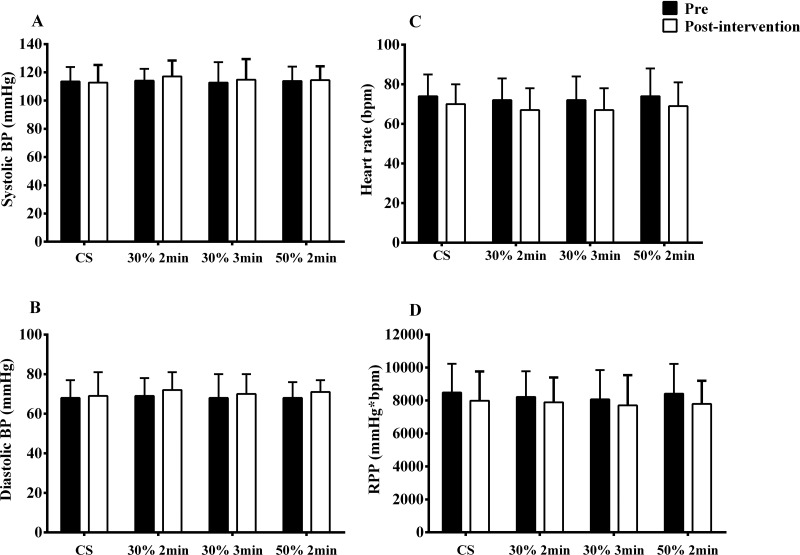
Systolic (A) and diastolic (B) blood pressure (BP), heart rate (C), and rate-pressure product (RPP - D) before and after a session of handgrip exercise with different intensities and volumes. CS - control session.

No differences were found in pre- to post-exercise comparisons for all cardiovascular parameters among sessions (*p*>0.05) (Figure 2). The mean response net effect revealed small and similar changes for systolic blood pressure (S30%2min: 3.8±7.6 mmHg; S30%3min: 2.8±9.0 mmHg; S50%2min: 1.4±8.4 mmHg) and diastolic blood pressure (S30%2min: 1.8±5.1 mmHg; S30%3min: 1.7±5.1 mmHg; S50%2min: 0.2±6.0 mmHg).

**Figure 3:**
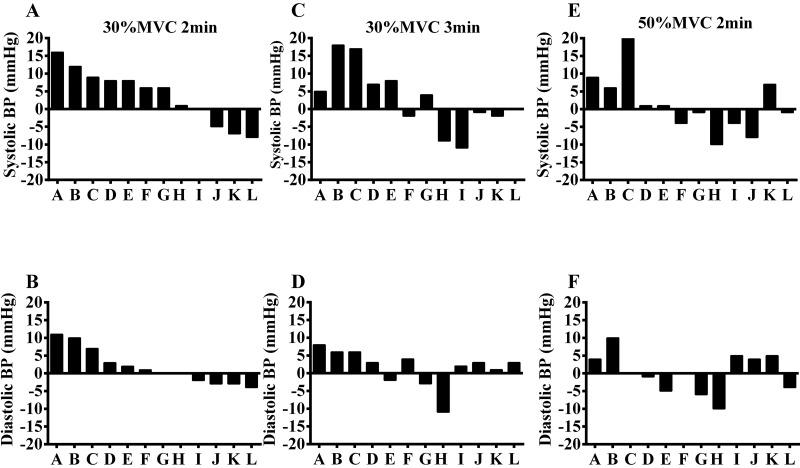
Individual net effect of the systolic and diastolic blood pressure (BP) after a session of exercise with different intensities and volumes.

Figure 3 shows the net effect of individual responses (corrected by the changes in the CS) for systolic and diastolic blood pressure. Individual analysis revealed high heterogeneity in the responses after the IHG protocol. Eight of the 12 patients exhibited systolic or diastolic blood pressure reductions ≥4.0 mmHg in at least one session. Following the S30%2min session, three of 12 patients exhibited reductions ≥4.0 mmHg in systolic blood pressure, while one patient exhibited this change for diastolic blood pressure. Two patients exhibited reductions ≥4.0 mmHg in systolic blood pressure, and one patient exhibited a reduction ≥4.0 mmHg in diastolic blood pressure in the S30%3min session. Four patients exhibited reductions ≥4.0 mmHg systolic blood pressure and four patients had reductions ≥4.0 mmHg for diastolic blood pressure in the S50%2min session. In addition, among those who had reductions ≥4.0 mmHg in blood pressure, the greatest reductions were observed in the S50%2min session (62.5% of patients).

Seven patients exhibited increases ≥4.0 mmHg in systolic or diastolic blood pressure in one of the sessions. Three patients exhibited increased systolic and diastolic blood pressure after all sessions, with values varying between 5 and 18 mmHg for systolic blood pressure and between 4 and 10 mmHg for diastolic blood pressure. In addition, 57.1% of the patients who exhibited increases in blood pressure ≥4.0 mmHg showed the greatest increases after the S30%2min session.

Table 3 shows a comparison of the clinical characteristics between patients who exhibited (n=7) or did not exhibit (n=5) reductions in blood pressure ≥4.0 mmHg. Patients who exhibited reduced blood pressure after an IHG session had a higher body mass index, diastolic blood pressure and heart rate (*p*<0.05), and they tended to be younger (*p*=0.07).

## DISCUSSION

The primary finding of the present study is that different IHG protocols did not reduce any of the measured cardiovascular parameters. Analyses of the individual data demonstrated heterogeneity in responses, including increases in blood pressure during some sessions. Patients with higher body mass index, diastolic blood pressure and heart rate were more likely to experience decreases in blood pressure ≥4 mmHg during at least one exercise session.

The results of the present study indicated that IHG exercise did not acutely alter the systolic or diastolic blood pressure, heart rate or rate-pressure product of hypertensive patients. Although acute reductions in blood pressure after exercise were shown in several studies 14,22, this effect is not a universal finding. Similar to the present study, Ash et al. 23 and Goessler et al. 24 did not observe changes in cardiovascular variables after a single IHG session in pre-hypertensive obese subjects or patients with coronary artery disease, respectively. Taken together these data suggest that IHG exercise generally produced no acute effects on cardiovascular parameters in patients with different conditions 23,24.

Patients in the current study had well-controlled cardiovascular parameters (∼113/68 mmHg; 74 bpm; 8487 mmHg*bpm), which may be associated with the fact that these patients had already performed six weeks of isometric exercise training, which was sufficient to reduce blood pressure. Trained hypertensive patients are less responsive to acute effects 25,26. Acute reductions in blood pressure after exercise are also attenuated in subjects with low initial blood pressure 27. Individual data analysis demonstrated different acute cardiovascular responses after sessions. Notably, systolic blood pressure was increased in 67%, 50% and 50% of patients after the S30%2min, S30%3min and S50%2min sessions, respectively. This report is the first study to present individual data of acute responses to isometric exercise, and the results indicate the existence of responders and non-responders after isometric exercise sessions.

Sample characteristics may explain the differences between responder and non-responder. Patients with reductions in blood pressure had a higher body mass index, diastolic blood pressure and heart rate than patients who did not exhibit reductions. These factors affect cardiovascular responses following exercise 27,28, and it is possible that greater impairment in a patient's cardiac and inflammatory profiles increases the occurrence of acute benefits after an IHG session.

Notably, the greatest acute decreases in blood pressure occurred after the higher intensity session (approximately 60% of patients). The mechanisms underlying this response were not assessed. However, the mechanical contraction of the muscles around the vessels during the S50%2min session may have produced a higher occlusion of blood flow than the lower intensity exercise and increased shear stress, which stimulated nitric oxide production and vasodilation. Millar et al. 29 suggested that the systolic blood pressure responses after IHG exercises were related to mechanoreceptor stimulation during exercise. Therefore, contraction intensity may be a determining factor of cardiovascular responses. Another study 30 observed that local tissue hypoxia during IHG exercise was involved in the metabolic attenuation of sympathetic vasoconstriction in the microcirculation of active muscle. Therefore, lower intensity in IHG sessions did not evoke metaboreflex responses or large changes in skeletal muscle metabolites 31, which prevented a decrease in blood pressure.

IHG exercise performed with different intensities and volumes did not reduce blood pressure in trained hypertensive patients. The chronic effects of IHG exercise on clinical blood pressure are widely demonstrated in the literature 4,6,29,32, but the acute effects remain uncertain, especially in controlled and trained hypertensive patients.

This study has some limitations that should be considered. The small sample size precluded us from performing additional analyses, primarily to compare the clinical conditions. The specific characteristics of the sample population may limit the generalizability of the results to other populations that differ in age, sex, or health status. We included men and women, and the influence of sex differences on the mechanisms underlying the cardiovascular responses to exercise cannot be excluded. The patients were monitored for only 30 min, and whether the post-exercise duration influenced the results was not examined in this study.

In conclusion, IHG exercise performed with different intensities and volumes did not reduce the blood pressure of controlled and trained hypertensive patients.

## AUTHOR CONTRIBUTIONS

Silva GO, Farah BQ and Ritti-Dias RM were responsible for the study conception and design, analysis and interpretation of data, draft of the manuscript, critical revision of the manuscript for important intellectual content and approval of the final version to be published. Germano-Soares AH, Andrade-Lima A, Santana FS and Rodrigues SL were responsible for the manuscript drafting, critical revision of the manuscript for important intellectual content and approval of the final version to be published.

## Figures and Tables

**Table 1:** General characteristics of the hypertensive patients included in this study.

Variables	Values
Age (years)	58.5±6
Weight (kg)	84.2±16.4
Height (m)	1.63±0.08
Body mass index (kg/m^2^)	31.5±5.7
Maximum voluntary contraction (kgf)	29.7±10.3
**Medications**	
β-blocker (%)	42
Calcium channel blocker (%)	17
Angiotensin-converting enzyme inhibitor (%)	8
Angiotensin receptor antagonist (%)	67
Diuretic (%)	58

Data are presented as the means and standard deviations or relative frequencies.

**Table 2:** Comparisons of the hemodynamic parameters in the pre-intervention period among experimental sessions.

	Control	S30%2min	S50%2min	S30%3min	*p* value
Systolic blood pressure (mmHg)	114±10	114±8	114±10	113±14	0.94
Diastolic blood pressure (mmHg)	68±9	69±9	68±8	68±12	0.87
Heart rate (bpm)	74±11	72±11	74±14	71±12	0.38
Rate-pressure product (mmHg*bpm)	8485±1745	8214±1563	8414±1801	8071±1781	0.27

Data are presented as the means and standard deviations.

**Table 3:** Clinical characteristics of patients who exhibited a reduction ≥4.0 mmHg in at least one of the types of exercise (n=7) compared to patients who did not exhibit this response (n=5).

Variables	Patients who did not exhibit reductions (≥4.0 mmHg)	Patients who exhibited reductions (≥4.0 mmHg)	*p*
Age (years)	62±6	56±5	0.07
Women (%)	20	29	0.99
Body mass index (kg/m^2^)	27±2	35±5	0.01*
Systolic blood pressure (mmHg)	111±6	116±12	0.39
Diastolic blood pressure (mmHg)	61±6	72±7	0.02*
Heart Rate (bpm)	64±10	79±11	0.04*
Maximum voluntary contraction (kgf)	28±12	31±9	0.59
*Medications*			
β-blocker (%)	40	43	0.99
Calcium channel blocker (%)	20	14	0.99
Angiotensin-converting enzyme inhibitor (%)	0	14	0.99
Angiotensin receptor antagonist (%)	60	71	0.99
Diuretic (%)	40	71	0.56

Data are presented as the means ± standard deviation; *Different from patients who did not exhibit reductions.

## References

[1] World Health Organization (2011). Global status report on noncommunicable diseases 2010.

[2] Lopez AD, Mathers CD, Ezzati M, Jamison DT, Murray CJ (2006). Global and regional burden of disease and risk factors, 2001: systematic analysis of population health data. Lancet.

[3] Lewington S, Clarke R, Qizilbash N, Peto R, Collins R (2002). Prospective Studies Collaboration. Age specific relevance of usual blood pressure to vascular mortality: a meta-analysis of individual data for one million adults in 61 prospective studies. Lancet.

[4] Badrov MB, Bartol CL, DiBartolomeo MA, Millar PJ, McNevin NH, McGowan CL (2013). Effects of isometric handgrip training dose on resting blood pressure and resistance vessel endothelial function in normotensive women. Eur J Appl Physiol.

[5] Millar PJ, Bray SR, MacDonald MJ, McCartney N (2008). The hypotensive effects of isometric handgrip training using an inexpensive spring handgrip training device. J Cardiopulm Rehabil Prev.

[6] Ray CA, Carrasco DI (2000). Isometric handgrip training reduces arterial pressure at rest without changes in sympathetic nerve activity. Am J Physiol Heart Circ Physiol.

[7] Wiley RL, Dunn CL, Cox RH, Hueppchen NA, Scott MS (1992). Isometric exercise training lowers resting blood pressure. Med Sci Sports Exerc.

[8] Carlson DJ, Inder J, Palanisamy SK, McFarlane JR, Dieberg G, Smart NA (2016). The efficacy of isometric resistance training utilizing handgrip exercise for blood pressure management: a randomized trial. Medicine (Baltimore).

[9] Farah BQ, Germano-Soares AH, Rodrigues SLC, Santos CX, Barbosa SS, Vianna LC (2017). Acute and Chronic Effects of Isometric Handgrip Exercise on Cardiovascular Variables in Hypertensive Patients: A Systematic Review. Sports (Basel).

[10] Brook RD, Appel LJ, Rubenfire M, Ogedegbe G, Bisognano JD, Elliott WJ (2013). American Heart Association Professional Education Committee of the Council for High Blood Pressure Research, Council on Cardiovascular and Stroke Nursing, Council on Epidemiology and Prevention, and Council on Nutrition, Physical Activity. Beyond medications and diet: alternative approaches to lowering blood pressure: a scientific statement from the american heart association. Hypertension.

[11] Anyfanti P, Triantafyllidou E, Papadopoulos S, Triantafyllou A, Nikolaidis MG, Kyparos A (2017). Smoking before isometric exercise amplifies myocardial stress and dysregulates barorreceptor sensitivity and cerebral oxygenation. J Am Soc Hypertens.

[12] Atkinson CL, Carter HH, Naylor LH, Dawson EA, Marusic P, Hering D (2015). Opposing effects of shear-mediated dilation and myogenic constriction on artery diameter in response to handgrip exercise in humans. J Appl Physiol (1985).

[13] Olher Rdos R, Bocalini DS, Bacurau RF, Rodriguez D, Figueira A, Pontes FL (2013). Isometric handgrip does not elicit cardiovascular overload or post-exercise hypotension in hypertensive older women. Clin Interv Aging.

[14] van Assche T, Buys R, de Jaeger M, Coeckelberghs E, Cornelissen VA (2017). One single bout of low-intensity isometric handgrip exercise reduces blood pressure in healthy pre- and hypertensive individuals. J Sports Med Phys Fitness.

[15] Moreira SR, Cucato GG, Terra DF, Ritti-Dias RM (2016). Acute blood pressure changes are related to chronic effects of resistance exercise in medicated hypertensives elderly women. Clin Physiol Funct Imaging.

[16] Lima AH, Miranda AS, Correia MA, Soares AH, Cucato GG, Sobral Filho DC (2015). Individual blood pressure responses to walking and resistance exercise in peripheral artery disease patients: Are the mean values describing what is happening?. J Vasc Nurs.

[17] Costa EC, Dantas TC, de Farias Junior LF, Frazão DT, Prestes J, Moreira SR (2016). Inter- and Intra-Individual Analysis of Post-Exercise Hypotension Following a Single Bout of High-Intensity Interval Exercise and Continuous Exercise: A Pilot Study. Int J Sports Med.

[18] Farah BQ, Correia MA, Rodrigues SL, Cavalcante BR, Ritti-Dias RM (2014). Reliability of handgrip maximal voluntary contraction in hypertensive adults. Braz J Phys Act Health.

[19] Fess E, Casanova JS (1992). Grip strength. Clinical assessment recommendations.

[20] Sociedade Brasileira de Cardiologia; Sociedade Brasileira de Hipertensão; Sociedade Brasileira de Nefrologia (2010). [VI Brazilian Guidelines on Hypertension]. Arq Bras Cardiol.

[21] Touyz RM, Campbell N, Logan A, Gledhill N, Petrella R, Padwal R, Canadian Hypertension Education Program (2004). The 2004 Canadian recommendations for the management of hypertension: Part III--Lifestyle modifications to prevent and control hypertension. Can J Cardiol.

[22] Souza LR, Vicente JB, Melo GR, Moraes VC, Olher RR, Sousa IC (2018). Acute Hypotension after Moderate-Intensity Handgrip Exercise in Hypertensive Elderly People. J Strength Cond Res.

[23] Ash GI, Taylor BA, Thompson PD, MacDonald HV, Lamberti L, Chen MH (2017). The antihypertensive effects of aerobic versus isometric handgrip resistance exercise. J Hypertens.

[24] Goessler K, Buys R, Cornelissen VA (2016). Low-intensity isometric handgrip exercise has no transient effect on blood pressure in patients with coronary artery disease. J Am Soc Hypertens.

[25] Moraes MR, Bacurau RF, Simões HG, Campbell CS, Pudo MA, Wasinski F (2012). Effect of 12 weeks of resistance exercise on post-exercise hypotension in stage 1 hypertensive individuals. J Hum Hypertens.

[26] Mota MR, Oliveira RJ, Terra DF, Pardono E, Dutra MT, de Almeida JA (2013). Acute and chronic effects of resistance exercise on blood pressure in elderly women and the possible influence of ACE I/D polymorphism. Int J Gen Med.

[27] Forjaz CL, Tinucci T, Ortega KC, Santaella DF, Mion D, Negrão CE (2000). Factors affecting post-exercise hypotension in normotensive and hypertensive humans. Blood Press Monit.

[28] Brito LC, Queiroz AC, Forjaz CL (2014). Influence of population and exercise protocol characteristics on hemodynamic determinants of post-aerobic exercise hypotension. Braz J Med Biol Res.

[29] Millar PJ, MacDonald MJ, McCartney N (2011). Effects of isometric handgrip protocol on blood pressure and neurocardiac modulation. Int J Sports Med.

[30] Hansen J, Sander M, Hald CF, Victor RG, Thomas GD (2000). Metabolic modulation of sympathetic vasoconstriction in human skeletal muscle: role of tissue hypoxia. J Physiol.

[31] Batman BA, Hardy JC, Leuenberger UA, Smith MB, Yang QX, Sinoway LI (1994). Sympathetic nerve activity during prolonged rhythmic forearm exercise. J Appl Physiol (1985).

[32] Inder JD, Carlson DJ, Dieberg G, McFarlane JR, Hess NC, Smart NA (2016). Isometric exercise training for blood pressure management: a systematic review and meta-analysis to optimize benefit. Hypertens Res.

